# Editorial: Bioactive substances-mediated targeted therapy of cardio-cerebrovascular diseases

**DOI:** 10.3389/fphar.2023.1146426

**Published:** 2023-02-02

**Authors:** Xiaoyong Tong, Yi Tan, Linxi Chen, Guixue Wang

**Affiliations:** ^1^ School of Pharmaceutical Sciences, Chongqing University, Chongqing, China; ^2^ University of Louisville School of Medicine, Louisville, KY, United States; ^3^ Institute of Pharmacy and Pharmacology, University of South China, Hengyang, Hunan, China; ^4^ Key Laboratory of Biorheological and Technology of Ministry of Education, State and Local Joint Engineering Laboratory for Vascular Implants, Modern Life Science Experiment Teaching Center at Bioengineering College of Chongqing University, Chongqing, China

**Keywords:** cardio-cerebrovascular diseases, bio-nanotechnology, bioactive substance, Traditional Chinese Medicine, targeted drug delivery

Cardio-cerebrovascular diseases (CVDs), one of the leading causes of death worldwide, are a broad spectrum of serious health conditions including heart disease, hypertension, stroke, atherosclerosis, retinopathy, etc. The pathogenesis and clinical features of CVDs have been well-studied in the past decades. However, precision medicine which leaves out normal tissue and only targets pathogenic molecules or cells is limited. The currently available or FDA-approved drugs are insufficient to treat most of these diseases, so there is an urgent need for new candidate drugs and/or strategies for drug delivery.

The rapid advances in bio-nanotechnology and bioactive substances allow the feasibility of the design and implementation of strategies for targeted drug delivery to treat CVDs and the invention of cardio-cerebrovascular regenerative medicine. Compared with newly discovered drugs, the active ingredients and derivatives of Traditional Chinese Medicine (TCM) have attracted more attention in CVD treatment as they involve lower risk, possible reduction of expenditure and shorter development time for they have been sold and tested on the market for a long time.

The Research Topic “Bioactive substances-mediated targeted therapy of cardio-cerebrovascular diseases” aims to create a forum for current advances in preclinical and clinical studies utilizing bioactive substances materials and active components of TCM and related composites, to design and develop therapies that specifically target molecular and cellular pathogenesis of CVD to support, enhance, or replace damaged CVD tissues or biological functions while focusing on the topics within the scope of the journal. This topic is led by the above four Guest Editors, who are experts on this topic and supervise the whole editing process of submitting papers. A total of eleven articles were published, including eight original research and three review articles.

Ferroptosis is closely related to cardiomyocyte death. Clioquinol can inhibit ferroptosis. Li et al. used porous lipid-poly (lactic-co-glycolic acid) microbubbles as carriers to deliver clioquinol, which showed the advantages of high drug loading, good biocompatibility, sustained release, improved effect of clioquinol and reduced its cytotoxicity as tested in cardiomyocytes.

Bhattacharjee et al. reported that the phenolic compounds in Garcinia pedunculata extract protected against isoproterenol-induced rat cardiac hypertrophy by reducing oxidative stress and inflammation, suggesting the cardioprotective potential of Garcinia pedunculata.

Lv et al. reported that Icariside II, a flavonol glycoside derived from the TCM Herba Epimedii, suppressed vascular smooth muscle cell phenotypic transition by modulating the focal adhesion signaling pathway, and improved vascular remodeling in a rat’s balloon injury model. Their research emphasizes the therapeutic efficacy and underlying mechanisms of Icariside II on vascular remodeling.

Dan-Shen-Yin is a TCM used in the treatment of CVDs. Endothelial-to-mesenchymal transition plays an important role in the pathogenesis of atherosclerosis. Hong et al. employed a network pharmacology-based strategy and found that Dan-Shen-Yin could inhibit endothelial-to-mesenchymal transition by suppressing the integrin/PI3K/AKT signaling pathway, proposing a potential therapeutic intervention for atherosclerosis.

Rhizoma Corydalis is clinically used to treat myocardial infarction in China. By network pharmacology and experimental verification, Li et al. reported that Rhizoma Corydalis protected against myocardial infarction by activating the PI3K/AKT pathway, providing a scientific basis for Rhizoma Corydalis in treating myocardial infarction.

In a transverse aortic constriction mouse model, Zhang et al. found that a (Pro)renin receptor decoy inhibitor PRO20 retarded cardiac remodeling and heart failure by increasing cAMP levels and reducing endoplasmic reticulum stress in both cardiomyocytes and cardiac fibroblasts.

Safflower is an important TCM for promoting blood circulation and removing blood stasis. Wang et al. reported that its bioactive compound 6-hydroxykaempferol 3,6-di-O-glucoside-7-O-glucuronide protected against endothelial injury by regulating expressions of hypoxia-inducible factor-1 alpha and nuclear factor kappa B, and exhibited anti-thrombotic activity in phenylhydrazine-induced zebrafish thrombosis model.

Obesity is a potential risk factor for CVDs. Jiang et al. used transcriptomic analyses to identify genes associated with the regulation of myogenic differentiation and found that apolipoprotein L 9a regulated myogenic differentiation by activating the ERK1/2 pathway, which provides a promising therapeutic target for intervention in obesity-induced muscle atrophy.

In a review article, Yang et al. discussed the drug delivery system based on nanoparticles for CVD treatment. The drug delivery system based on nanoparticles changes the biological distribution of therapeutic agents through targeted delivery and controlled drug release of precise drugs. Nanoparticles based on metals, lipids and polymers are ideal materials for CVD treatment. In addition, this review also discusses the potential role of nanoparticles in metabolism and post-therapeutic toxicity.

Macrophage is a major contributor to atherosclerosis progression, and its associated pathological process is an important target for diagnosing and treating atherosclerosis. In a review article, Hu et al. specifically summarized the macrophage-targeted nanomedicine for the diagnosis and management of atherosclerosis, their potential applications and clinical benefits.

Stem cell-based therapies have emerged as promising treatment options for CVDs. In a mini review, Ma et al. summarized the current means of treatment of long non-coding RNAs from stem cell-derived exosomes for CVDs and discussed the current challenges and prospects of long non-coding RNAs treatment for CVDs.

Despite progress in the management of CVDs, drug treatment is still not ideal due to poor pharmacokinetics and high toxicity. This Research Topic has an in-depth understanding of the latest research findings and updates related to the ongoing strategy and drug discovery research in various treatment areas of current interest, including technological progress and challenges. The rapid development of biological nanotechnology, bioactive substances as well as the development of active ingredients and derivatives of TCM have received more attention, however, challenges lie ahead. Key assessments of preclinical, clinical and observational data/evidence are needed to investigate candidate drug efficacy and safety/toxicity.

